# Efficacy and Safety of Traditional Chinese Medicine Retention Enema for Endometriosis: A Systematic Review and Meta-Analysis

**DOI:** 10.3390/ph19020279

**Published:** 2026-02-06

**Authors:** Na-Yoen Kwon, Eun-Jin Kim, Yong-Taek Oh, Soo-Hyun Sung, Hyun-Kyung Sung

**Affiliations:** 1 Department of Obstetrics and Gynecology, College of Korean Medicine, Ga-Chon University, Seongnam-si 13120, Republic of Korea; kwonnay@gachon.ac.kr; 2 Department of Pediatrics of Korean Medicine, Korean Medicine Hospital, Dongguk University Bundang Medical Center, Seongnam-si 13601, Republic of Korea; utopialimpid@naver.com; 3 Department of Korean Medicine, Woosuk University, Jeonju 55338, Republic of Korea; ydydxor@gmail.com; 4 Department of Policy Development, National Institute for Korean Medicine Development, Seoul 04516, Republic of Korea; 5 Department of Education, College of Korean Medicine, Dongguk University, Gyeongju-si 38066, Republic of Korea

**Keywords:** endometriosis, retention enema therapy, herbal medicine, systematic review, meta-analysis

## Abstract

**Background/Objectives**: Herbal retention enema is widely used in Traditional Chinese Medicine for the management of endometriosis; however, its clinical efficacy and safety have not been systematically evaluated. **Methods**: Ten electronic databases were searched up to May 2025 to identify randomized controlled trials evaluating herbal retention enema as a standalone treatment for endometriosis. Primary outcomes included total effective rate and pain-related measures. Risk of bias was assessed using the Cochrane RoB 2 tool. **Results**: Nine randomized controlled trials involving 614 participants were included. Compared with Western medical therapies, herbal retention enema was associated with greater overall clinical effectiveness (OR = 2.87) and significant improvement in dysmenorrhea. When compared with oral herbal medicine, herbal retention enema demonstrated a higher total effective rate (OR = 7.13). A trend toward improved pregnancy outcomes was observed (*p* = 0.05). No serious adverse events related to herbal retention enema were reported, and systemic adverse effects were less frequent than with hormonal therapies. **Conclusions**: Herbal retention enema may represent a potential complementary option for symptom management in endometriosis; however, the certainty of evidence remains low due to important methodological limitations in the included trials. However, given methodological limitations and heterogeneity among studies, further high-quality randomized controlled trials with standardized outcomes and long-term follow-up are required.

## 1. Introduction

Endometriosis is a chronic, estrogen-dependent inflammatory condition characterized by the ectopic presence of endometrial-like tissue outside the uterine cavity. This ectopic endometrial tissue undergoes cyclical changes as normal endometrium, including bleeding, proliferation, secretion, and shedding in response to hormonal fluctuations throughout the menstrual cycle. However, bleeding from this area cannot be discharged, leading to local accumulation, chronic inflammation, fibrosis, and pathological adhesions to adjacent organs [1].

Endometriosis affects approximately 10% of women of reproductive age, causing pelvic pain, dysmenorrhea, dyspareunia and infertility [2,3]. Beyond physical symptoms, it significantly impairs quality of life and contributes to psychological stress and social dysfunction [3]. Delayed diagnosis, lack of definitive non-invasive biomarkers, and variable treatment responses make management challenging. In the United States, the annual socioeconomic cost of endometriosis has been estimated to exceed $22 billion, reflecting its substantial medical and societal burden [4].

The current standard treatment options for endometriosis include hormonal therapy and surgical intervention. Hormonal therapy—such as oral contraceptives, progestins, gonadotropin-releasing hormone agonists (GnRH-as), and aromatase inhibitors—is primarily aimed at suppressing ovulation and menstruation to induce a hypoestrogenic state and thereby reduce the stimulation of ectopic endometrial lesions.

Although these pharmacologic approaches can effectively alleviate symptoms in the short term, they are frequently associated with significant side effects, including vasomotor instability, mood disturbances, decreased bone mineral density, and weight gain. Furthermore, recurrence rates following cessation of hormonal treatment remain high, often exceeding 50% within five years [5,6].

Surgical management, including laparoscopic excision or ablation of endometriotic lesions and adhesiolysis, can offer symptomatic relief and may improve fertility outcomes in select patients. However, surgery is inherently invasive, carries operative risks, and does not guarantee permanent resolution. Recurrence of symptoms post-surgery is also common, and repeated surgeries may further compromise reproductive anatomy and ovarian reserve [6,7].

Given these limitations, there is growing interest in complementary and alternative therapies as potential adjunct or stand-alone interventions for managing endometriosis. Recent systematic reviews have demonstrated the potential of interventions such as acupuncture, Chinese herbal medicine, physical activity, and dietary modification to alleviate symptoms and improve quality of life in affected women [6,8,9,10]. These approaches offer a more holistic, patient-centered model of care with potentially fewer adverse effects and improved long-term tolerability.

Traditional Chinese Medicine (TCM), particularly herbal medicine, has drawn increasing attention as an adjunctive or alternative option for endometriosis management. Numerous clinical studies and systematic reviews have reported that Chinese herbal medicine may alleviate pelvic pain, reduce serum CA-125 levels, regulate estradiol (E2) levels, and improve fertility outcomes in women with endometriosis [6,11].

Herbal retention enema is a traditional TCM method involving the rectal infusion of decoctions maintained for extended durations. Herbal retention enema may offer unique pharmacokinetic and therapeutic advantages. Given the anatomical proximity of the rectum to the uterus and adnexa, this administration method allows for localized drug delivery, potentially enhancing the concentration of active compounds at the target site while bypassing first-pass hepatic metabolism. Moreover, it can reduce gastrointestinal irritation.

Herbal enemas have been widely studied in the treatment of chronic pelvic diseases, such as chronic prostatitis, chronic pelvic pain syndrome, and irritable bowel syndrome [12,13]. Recently, retention enema therapy has also been systematically evaluated in other gynecologic disorders, such as tubal obstructive infertility, where it demonstrated significant anti-inflammatory effects and the benefit of localized drug delivery to the pelvic cavity [14]. Despite these findings and its long-standing clinical use, no systematic review has yet synthesized the evidence specifically for endometriosis—a condition characterized by chronic pelvic inflammation.

While several systematic reviews have explored the efficacy of TCM for endometriosis, most have primarily focused on orally administered Chinese patent medicines or multimodal complementary interventions [6], making it difficult to discern the specific clinical impact of herbal retention enema. Unlike oral administration, herbal retention enema offers unique pharmacological advantages, such as bypassing hepatic first-pass metabolism and achieving higher local drug concentrations near pelvic lesions through rectal mucosal absorption. To the best of our knowledge, this is the first systematic review and meta-analysis to exclusively evaluate the efficacy and safety of TCM herbal retention enema as a standalone intervention for endometriosis. This study aims to address this gap by providing a comprehensive synthesis of current clinical evidence to inform future research and clinical decision-making.

This study therefore aims to fill this knowledge gap by systematically reviewing and analyzing the clinical efficacy and safety of herbal retention enema in the treatment of endometriosis-related symptoms.

## 2. Materials and Methods

### 2.1. Study Design

This study was conducted as a systematic review and meta-analysis to evaluate the efficacy and safety of Traditional Chinese Medicine retention enema in the management of endometriosis. The review process was performed in accordance with the Preferred Reporting Items for Systematic Reviews and Meta-Analyses (PRISMA) 2020 guidelines [15]. The study protocol was prospectively registered in the International Prospective Register of Systematic Reviews (PROSPERO; registration number: CRD420251018203, Available from https://www.crd.york.ac.uk/PROSPERO/view/CRD420251018203, accessed on 26 April 2025).

### 2.2. Search Strategy

A comprehensive literature search was conducted to identify relevant studies evaluating the effects of Traditional Chinese Medicine retention enema for endometriosis. The following electronic databases were searched up to 31 May 2025: PubMed, EMBASE, the Cochrane Central Register of Controlled Trials (CENTRAL), CINAHL via EBSCO, China National Knowledge Infrastructure (CNKI), Wanfang Data, Korean Studies Information Service System (KISS), Research Information Sharing Service (RISS), Oriental Medicine Advanced Searching Integrated System (OASIS), Korea Citation Index (KCI), and the Korean Medical Database (KMbase).

The search strategy was developed using a combination of controlled vocabulary (e.g., MeSH and Emtree terms, where applicable) and free-text keywords related to endometriosis and retention enema therapy. Search terms covered disease-related concepts (e.g., endometriosis) and intervention-related concepts (e.g., retention enema, herbal enema, Traditional Chinese Medicine). Boolean operators (AND/OR) were applied to combine search terms, and database-specific filters were used to identify randomized controlled trials.

The complete electronic search equations, including all database-specific Boolean operators and full search syntaxes, are provided in Supplementary File S1 to ensure transparency and reproducibility. No restrictions were applied regarding publication status. Studies published in English or Chinese were considered eligible. In addition, the reference lists of all included articles and relevant review papers were manually screened to identify additional eligible studies.

### 2.3. Eligibility Criteria

#### 2.3.1. Types of Studies

This review included randomized controlled trials (RCTs) evaluating the clinical effects of Traditional Chinese Medicine retention enema for the treatment of endometriosis. Non-randomized studies, observational studies, case reports, reviews, experimental studies (*in vivo* or *in vitro*), and study protocols were excluded.

#### 2.3.2. Types of Participants

Eligible participants were women diagnosed with endometriosis, regardless of age, disease stage, or diagnostic criteria.

#### 2.3.3. Types of Intervention and Comparators

The experimental group received Traditional Chinese Medicine retention enema administered as the sole therapeutic intervention. Studies evaluating retention enema combined with other active treatments, such as acupuncture, oral herbal medicine, moxibustion, or pharmacologic therapy, were excluded. The control group received a placebo, no treatment, or conventional therapy without retention enema.

#### 2.3.4. Types of Outcome Measures

Studies reporting at least one of the following outcomes were eligible for inclusion: pain-related outcomes (e.g., dysmenorrhea pain score), serum CA-125 levels, pregnancy rate, clinical effectiveness, recurrence rate, or adverse events.

Clinical effectiveness was primarily assessed using the total effective rate, a composite outcome frequently reported in Chinese trials. However, because this measure is not internationally standardized and its definition may vary across studies, pooled estimates based on total effective rate should be interpreted with caution.

### 2.4. Data Extraction

The identification of records was followed by their importation into EndNote 21 (Clarivate, Philadelphia, PA, USA), a process that facilitated the deduplication of all identified records. Two independent reviewers conducted a preliminary evaluation of the titles and abstracts to ascertain their relevance to the study. Subsequently, the reviewers conducted a full-text screening of the potentially relevant studies. Discrepancies were resolved through discussion, and a third reviewer was consulted when necessary.

### 2.5. Risk of Bias

The risk of bias of the included randomized controlled trials was independently assessed by two reviewers using the Cochrane Risk of Bias tool, version 2 (RoB 2) [16]. Disagreements between reviewers were resolved through discussion, and a third reviewer was consulted when consensus could not be reached.

The RoB 2 tool evaluates five domains of potential bias: (1) bias arising from the randomization process, (2) bias due to deviations from intended interventions, (3) bias due to missing outcome data, (4) bias in measurement of the outcome, and (5) bias in selection of the reported result. Each domain was judged as “low risk of bias,” “some concerns,” or “high risk of bias” according to the signaling questions and decision algorithms provided by the RoB 2 guidance.

An overall risk of bias judgment was assigned to each study based on the highest risk level identified across the five domains. The results of the risk of bias assessment were summarized graphically and narratively.

### 2.6. Statistical Analysis

Meta-analyses were conducted when at least two studies reported the same outcome with comparable definitions and measurement methods. For continuous outcomes, pooled effect sizes were calculated using mean difference (MD) or standardized mean difference (SMD) with 95% confidence intervals (CIs), depending on the consistency of outcome measurement scales across studies. For dichotomous outcomes, risk ratios (RRs) with 95% CIs were calculated. Statistical heterogeneity among studies was assessed using the I^2^ statistic, with values of 25%, 50%, and 75% indicating low, moderate, and high heterogeneity, respectively. A random-effects model was applied when substantial heterogeneity was detected (I^2^ > 50%); otherwise, a fixed-effect model was used.

When meta-analysis was not feasible due to heterogeneity or insufficient data, results were summarized using a narrative synthesis. Publication bias was not formally assessed using funnel plots or statistical tests when fewer than 10 studies were available for a given outcome. All statistical analyses were performed using Review Manager (RevMan), version 5.4 (The Cochrane Collaboration).

## 3. Results

### 3.1. Study Selection

A comprehensive literature search was conducted across 10 electronic databases, including PubMed, EMBASE, CINAHL via EBSCO, Web of Science, China National Knowledge Infrastructure (CNKI), Wanfang Data, Korean Studies Information Service System (KISS), Research Information Sharing Service (RISS), Oriental Medicine Advanced Searching Integrated System (OASIS), Korea Citation Index (KCI), and the Korean Medical Database (KMbase).

Among these sources, records were identified from PubMed (*n* = 42), EMBASE (*n* = 312), CINAHL (*n* = 27), Web of Science (*n* = 111), and CNKI (*n* = 177), yielding a total of 669 records. Searches conducted in the remaining databases did not identify additional eligible records relevant to the review topic.

After removal of 13 duplicate records, 656 records were screened based on titles and abstracts. During this screening stage, 369 records were excluded for failing to meet the predefined eligibility criteria. Consequently, 287 reports were sought for full-text retrieval.

**Figure 1:**
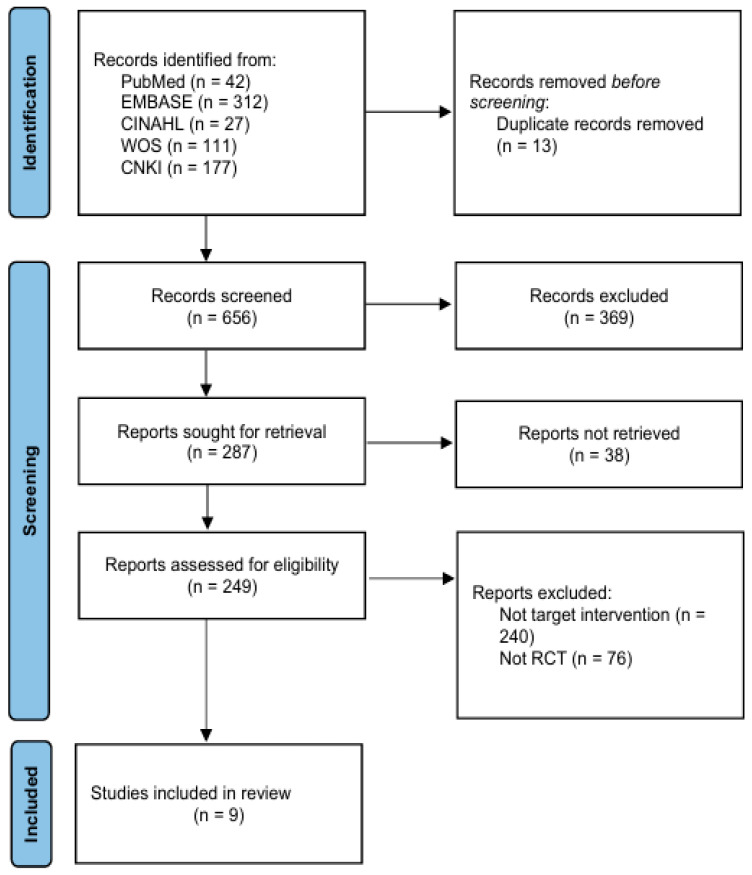
Flow chart of the review.

Of these, 38 reports could not be retrieved, and 249 full-text articles were assessed for eligibility. Following full-text evaluation, 240 articles were excluded because they did not investigate the target intervention, and an additional 76 articles were excluded due to non-randomized study designs. Ultimately, nine randomized controlled trials met the inclusion criteria and were included in the systematic review and meta-analysis. The study selection process is illustrated in Figure 1.

### 3.2. Characteristics of Included Studies

A total of nine randomized controlled trials involving 614 participants were included in this systematic review. All included studies were conducted in China and published between 2007 and 2022. The sample size of individual studies ranged from 42 to 82 participants.

In all studies, the experimental group received Traditional Chinese Medicine retention enema as the primary intervention, while the control group received placebo, no treatment, or conventional therapy without retention enema, including hormonal agents. The duration of treatment varied across studies, ranging from 3 months to 6 months, with follow-up periods reported in several studies. The main characteristics of the included studies are summarized in Table 1.

In studies using oral herbal medicine as a comparator, Li et al. (2018) [20] administered *Guizhi Fuling Capsules*, whereas Zeng et al. (2018) [23] used *Shaofu Zhuyu Decoction*, which consisted of *Angelica sinensis* (15 g), *Paeoniae Radix Rubra* (15 g), *Chuanxiong Rhizoma* (12 g), *Corydalis Rhizoma* (12 g), *Typhae Pollen* (10 g), *Trogopterorum Faeces* (10 g), *Myrrha* (10 g), *Zingiberis Rhizoma* (6 g), *Cinnamomi Cortex* (6 g), and *Foeniculi Fructus* (5 g).

### 3.3. Characteristics of Intervention

The characteristics of the herbal retention enema interventions used in the included studies are summarized in Table 2. Across all studies, the experimental intervention consisted of Traditional Chinese Medicine herbal decoctions administered via rectal retention enema, aiming to deliver the herbal components locally to the pelvic region.

Although the specific herbal formulations varied among studies, the intervention protocols shared several common features. Enema volumes ranged from 100 to 180 mL, with 100 mL being the most frequently reported volume. Herbal retention enemas were generally administered once daily, with individual retention times ranging from 30 to 240 min, with most studies adopting a minimum retention duration of at least 30 min. The treatment duration varied across studies, most commonly ranging from three menstrual cycles or month to 6 months. In several studies, the intervention was initiated after surgical treatment or commenced following the completion of menstruation, and treatment was typically suspended during the menstrual period.

The herbal prescriptions commonly included herbs traditionally associated with activating blood circulation, resolving blood stasis, alleviating pain, and reducing inflammatory processes, such as *Angelica sinensis* (Oliv.) Diels, *Paeonia lactiflora* Pall. or *Paeonia veitchii* Lynch, *Ligusticum chuanxiong* Hort., and *Corydalis yanhusuo* W.T.Wang. While the exact combinations and dosages differed, the overall therapeutic principles underlying the enema formulations were broadly consistent across studies.

Details regarding the specific herbal compositions, dosages, frequency of administration, retention time, total treatment duration, and additional procedural considerations are presented in Table 2.

### 3.4. Outcomes

#### 3.4.1. Clinical Effectiveness

##### Retention Enema Versus Western Medicine

**Figure 2:**
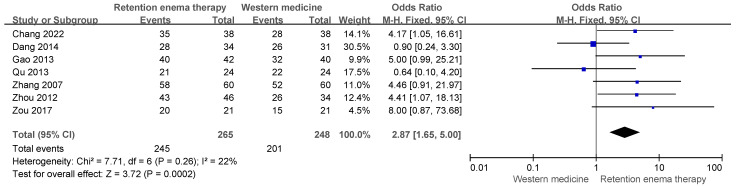
Forest plot of total effective rate comparing herbal retention enema with Western medicine. Blue squares indicate individual study estimates, and the black diamond indicates the pooled effect size. Studies included in this figure: Chang et al. (2022) [21], Dang et al. (2014) [19], Gao et al. (2013) [17], Qu et al. (2013) [18], Zhang et al. (2007) [24], Zhou et al. (2012) [22], Zou et al. (2017) [25].

Seven studies evaluated the total effective rate of herbal retention enema compared with Western medicine. Pooled analysis demonstrated that herbal retention enema significantly improved the total effective rate compared with Western medicine (OR = 2.87, 95% CI 1.65–5.00, *p* = 0.0002). Low heterogeneity was observed among the included studies (I^2^ = 22%) (Figure 2).

##### Retention Enema Versus Oral Herbal Medicine

**Figure 3:**
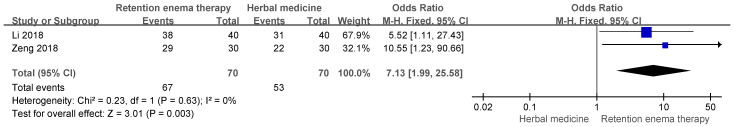
Forest plot of total effective rate comparing herbal retention enema with oral herbal medicine.Blue squares indicate individual study estimates, and the black diamond indicates the pooled effect size. Studies included in this figure: Li et al. (2018) [20], Zeng et al. (2018) [23].

Two studies compared herbal retention enema with oral herbal medicine in terms of total effective rate. Meta-analysis showed that herbal retention enema was associated with a significantly higher total effective rate compared with oral herbal medicine (OR = 7.13, 95% CI 1.99–25.58, *p* = 0.003), with no observed heterogeneity (I^2^ = 0%) (Figure 3).

#### 3.4.2. Pain-Related Outcomes

**Figure 4:**
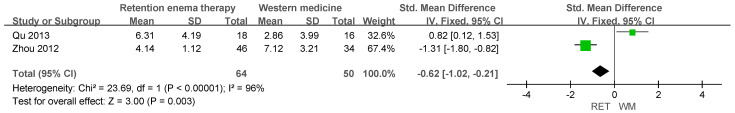
Forest plot of dysmenorrhea severity comparing herbal retention enema with Western medicine. Green squares indicate individual study estimates, and the black diamond indicates the pooled effect size. Studies included in this figure: Qu et al. (2013) [18], Zhou et al. (2012) [22].

Two studies reported dysmenorrhea-related pain scores and were included in the quantitative synthesis. Due to differences in pain assessment scales across studies, standardized mean difference (SMD) was used. Although the pooled analysis suggested a reduction in dysmenorrhea severity with herbal retention enema compared with Western medicine (SMD = −0.62, 95% CI −1.20 to −0.21, *p* = 0.003), the heterogeneity was extremely high (I^2^ = 96%), indicating major differences across studies. Therefore, this finding should be regarded as exploratory rather than definitive (Figure 4).

#### 3.4.3. Reproductive Outcomes

**Figure 5:**
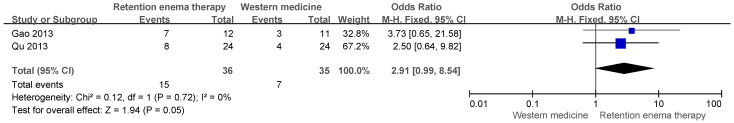
Forest plot of pregnancy rate comparing herbal retention enema with Western medicine. Blue squares indicate individual study estimates, and the black diamond indicates the pooled effect size. Studies included in this figure: Gao et al. (2013) [17], Qu et al. (2013) [18].

Two studies compared pregnancy rates between herbal retention enema and Western medicine. Pooled analysis indicated a higher pregnancy rate in the herbal retention enema group; however, the difference did not reach statistical significance (OR = 2.91, 95% CI 0.99–8.54, *p* = 0.05). No heterogeneity was detected (I^2^ = 0%) (Figure 5).

#### 3.4.4. Other Reported Outcomes

Several studies reported additional outcomes, including serum biomarkers and hormone levels such as CA-125, estradiol (E2), follicle-stimulating hormone (FSH), and inflammatory markers (e.g., TXB_2_, IL-1β, TNF-α). Due to the limited number of studies and heterogeneity in outcome definitions and measurement methods, these outcomes were summarized descriptively rather than quantitatively. Overall, the included studies consistently reported improvements in biomarker levels following herbal retention enema therapy compared with control interventions.

### 3.5. Adverse Events

Among the nine included randomized controlled trials, four studies did not report information on adverse events. The remaining five studies provided safety data for both the herbal retention enema and control groups and were included in a quantitative synthesis.

Across these studies, no serious adverse events related to herbal retention enema were reported. In the retention enema groups, reported adverse events were either absent or mild and transient, including occasional gastrointestinal discomfort such as abdominal distension or mild diarrhea, which resolved spontaneously without treatment discontinuation.

**Figure 6:**
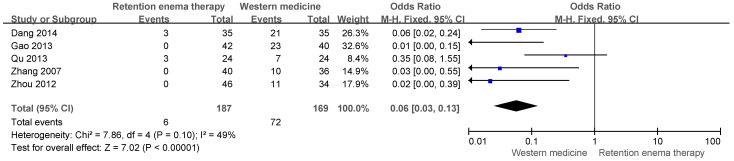
Forest plot of adverse event rate comparing herbal retention enema with Western medicine. Blue squares indicate individual study estimates, and the black diamond indicates the pooled effect size. Studies included in this figure: Dang et al. (2014) [19], Gao et al. (2013) [17], Qu et al. (2013) [18], Zhang et al. (2007) [24], Zhou et al. (2012) [22].

A meta-analysis of adverse event incidence demonstrated that herbal retention enema was associated with a significantly lower risk of adverse events compared with Western medicine (OR= 0.06, 95% CI 0.03–0.13, *p* < 0.00001), with moderate heterogeneity (I^2^ = 49%) (Figure 6). These findings suggest a more favorable tolerability profile for retention enema therapy, although adverse event reporting was incomplete across trials.

A summary of adverse events reported in individual studies is presented in Table 1.

### 3.6. Risk of Bias Assessment

**Figure 7:**
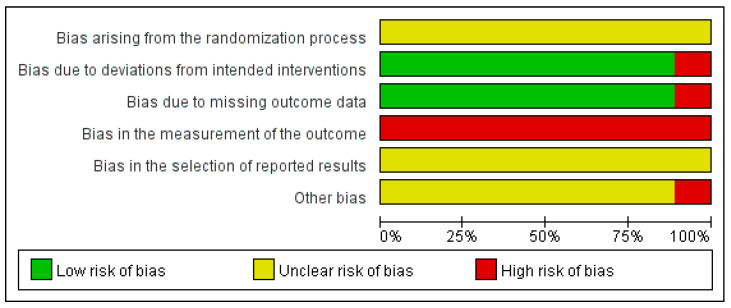
Risk of bias graph.

**Figure 8:**
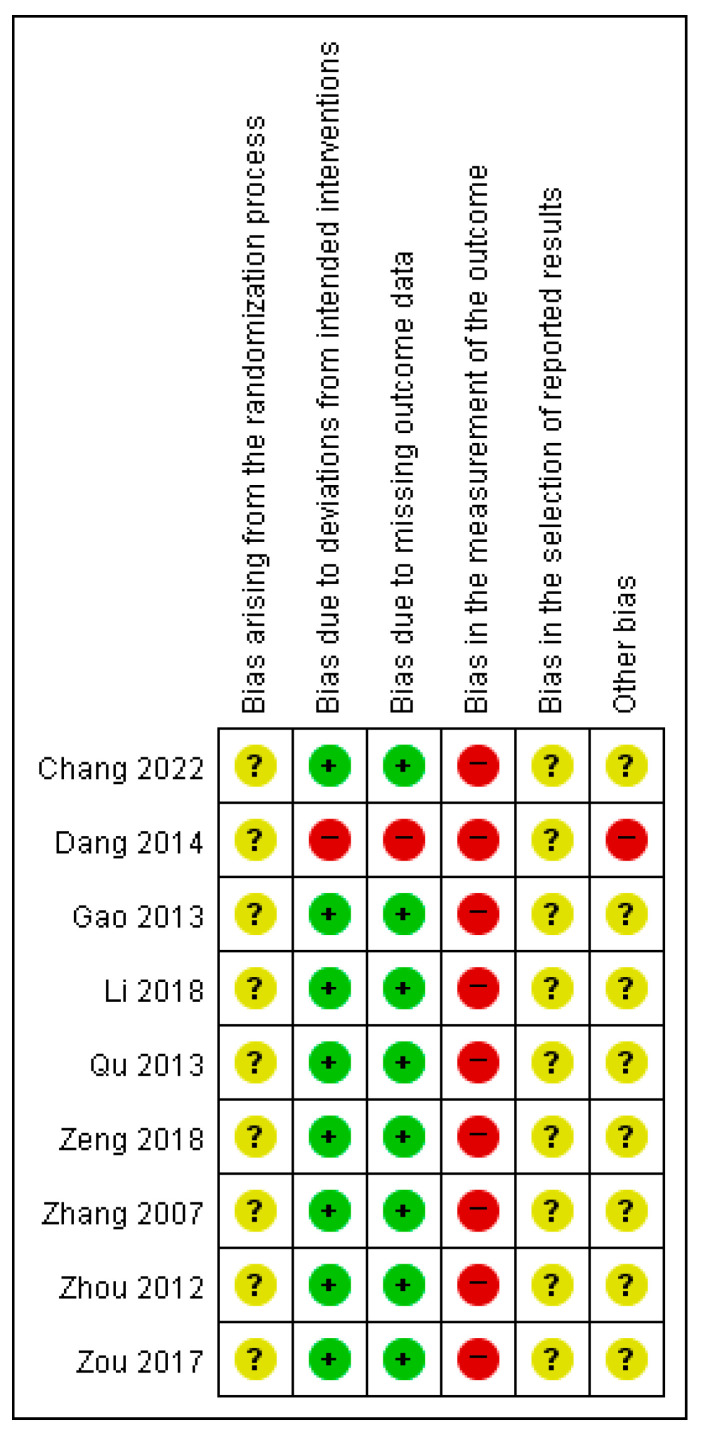
Risk of bias summary. Legend: “+” low risk of bias; “?” some concerns; “−” high risk of bias. Studies included in this figure: Chang et al. (2022) [21], Dang et al. (2014) [19], Gao et al. (2013) [17], Li et al. (2018) [20], Qu et al. (2013) [18], Zeng et al. (2018) [23], Zhang et al. (2007) [24], Zhou et al. (2012) [22], Zou et al. (2017) [25].

The risk of bias of the included studies was assessed using the Cochrane Risk of Bias tool, version 2 (RoB 2). The overall results of the risk of bias assessment are presented in Figure 7 (risk of bias graph) and Figure 8 (risk of bias summary).

Across the included studies, bias arising from the randomization process was judged as presenting some concerns in all trials, primarily due to insufficient reporting of random sequence generation and allocation concealment. No study was assessed as having a low risk of bias in this domain.

Regarding bias due to deviations from intended interventions and bias due to missing outcome data, most studies were judged to be at low risk of bias, although a small proportion of studies showed a high risk of bias due to lack of intention-to-treat analysis or incomplete outcome reporting.

In contrast, bias in the measurement of the outcome was assessed as high risk of bias in all included studies, mainly because outcomes such as pain scores and clinical effectiveness were subjectively assessed and outcome assessors were not blinded. Similarly, bias in the selection of reported results was judged as presenting some concerns or a high risk of bias in several studies due to the absence of prespecified protocols or trial registrations.

With respect to other bias, most included studies were assessed as having some concerns rather than a high risk of bias, mainly related to insufficient methodological reporting and unclear control of potential confounding factors.

Overall, the included studies were judged as having an overall risk of bias of some concerns, rather than high risk, with common methodological limitations observed across multiple domains.

## 4. Discussion

### 4.1. Principal Findings and Clinical Implications

This study represents the first systematic review and meta-analysis to comprehensively evaluate the clinical efficacy and safety of Traditional Chinese Medicine herbal retention enema in patients with endometriosis. Although herbal retention enema was associated with statistically significant improvements in clinical outcomes, these findings should be interpreted cautiously because the included trials exhibited important methodological limitations, particularly in randomization procedures and outcome assessment. Notably, the large effect size observed in comparison with oral herbal medicine (OR = 7.13) suggests that the route of administration, which allows delivery of herbal compounds to pelvic lesion–adjacent areas, may partly contribute to the observed clinical benefits.

With respect to pain-related outcomes, herbal retention enema demonstrated a statistically significant reduction in dysmenorrhea severity compared with Western medical treatment. However, variability in pain assessment tools and substantial inter-study heterogeneity were observed, indicating that caution is warranted when generalizing the magnitude of the analgesic effect. Given the extreme heterogeneity, the observed analgesic benefit cannot be considered robust, and differences in pain scales, herbal formulations, and treatment duration may have contributed substantially to between-study variability. Regarding reproductive outcomes, although the pooled analysis did not reach statistical significance for pregnancy rate (*p* = 0.05), a trend toward a higher pregnancy rate was observed in the retention enema group, suggesting a potential role in the management of endometriosis-associated infertility that warrants further investigation.

In terms of safety, no serious adverse events related to herbal retention enema were reported across the included studies. Mild and transient adverse events, such as gastrointestinal discomfort or abdominal distension, were occasionally reported but resolved without treatment discontinuation. In contrast, adverse events were more frequently reported in the Western medicine groups, including weight gain, abnormal liver function, acne, and irregular vaginal bleeding, which are commonly associated with systemic hormonal therapies.

Importantly, a pooled meta-analysis of adverse event incidence demonstrated that herbal retention enema was associated with a significantly lower risk of adverse events compared with Western medical therapies (OR = 0.06, 95% CI 0.03–0.13) (Figure 6), suggesting a more favorable tolerability profile. Nevertheless, because a quantitative meta-analysis of adverse event incidence was not conducted, these findings should be interpreted as indicating a relatively favorable tolerability profile, rather than definitive evidence of superior safety.

From a mechanistic perspective, the symptom improvements observed in this review are biologically plausible given the anti-inflammatory actions reported for several commonly used herbal components in retention enema formulations. Endometriosis is characterized by chronic pelvic inflammation and activation of cytokine-mediated pathways, including NF-κB signaling and elevated mediators such as TNF-α and prostaglandins [26]. Experimental studies have shown that Angelica sinensis–based preparations can downregulate inflammatory mediators, including COX-2, PGE2, and TNF-α, while also reducing oxidative stress markers [27]. In addition, paeoniflorin, a major constituent of Paeoniae Radix, has been reported to exert anti-inflammatory effects through modulation of NF-κB–related pathways, leading to decreased expression of IL-1β, IL-6, and TNF-α [28]. Curcumin, a representative compound derived from Curcuma species frequently included in these formulations, is also widely recognized as an NF-κB–centered anti-inflammatory modulator with downstream suppression of COX-2 and iNOS activity [29]. Collectively, these overlapping mechanisms provide a pharmacological rationale for the potential benefits of localized rectal delivery of multi-component herbal decoctions in alleviating inflammatory pain associated with endometriosis. Notably, these mechanistic findings are largely derived from non-endometriosis inflammatory models, and direct validation in endometriosis-specific systems remains necessary.

From a drug delivery perspective, the clinical benefits observed with herbal retention enema may also be explained by the pharmacokinetic advantages of rectal administration. The rectal route enables relatively constant local conditions with low enzymatic activity and can partially bypass hepatic first-pass metabolism, thereby improving the bioavailability of active compounds that are poorly absorbed or unstable when administered orally. In addition, rectal delivery allows retention of larger volumes and facilitates localized drug exposure in anatomically adjacent pelvic tissues, which may be particularly relevant in endometriosis as a chronic inflammatory disease confined to the pelvic cavity. Recent advances in rectal drug delivery systems further highlight the potential of this route for enhanced mucosal absorption, prolonged retention, and targeted distribution through novel carriers such as nanoparticles, liposomes, and thermosensitive hydrogels. These considerations support the biological plausibility that multi-component herbal decoctions administered via retention enema could achieve higher local concentrations near ectopic lesions while minimizing systemic adverse effects [30].

An additional observation from this review is that, despite heterogeneity in herbal formulations, several procedural characteristics of retention enema were reported consistently across studies. Reported enema volumes were generally sufficient to allow the herbal solution to reach the upper rectum, anatomically adjacent to the recto-uterine pouch, a common site of endometriotic lesions. Moreover, retention times of 30 min or longer were commonly adopted, which may increase mucosal contact time and local exposure while avoiding first-pass hepatic metabolism.

Although these procedural features cannot be regarded as standardized or optimized based on the current evidence, their consistent reporting across studies suggests that the mode of administration itself may play an important role in clinical outcomes. This observation underscores the need for future research to systematically examine procedural parameters and to develop standardized protocols for both clinical practice and research settings.

Overall, the findings of this meta-analysis indicate that herbal retention enema is one of the interventions with the most consistent evidence for alleviating pain and improving overall clinical symptoms in endometriosis. From a clinical perspective, herbal retention enema may be considered a complementary therapeutic option, particularly for patients who experience difficulty maintaining long-term hormonal therapy due to adverse effects or who require supportive management following surgical treatment. Further well-designed randomized controlled trials with standardized outcome measures and longer follow-up periods are needed to clarify its effects on reproductive outcomes and long-term prognosis.

### 4.2. Comparison with Previous Studies

Previous systematic reviews and meta-analyses on complementary and alternative medicine (CAM) for endometriosis have primarily evaluated acupuncture, oral traditional Chinese medicine (TCM), and dietary interventions. While these studies generally reported favorable outcomes for pain relief, most focused on systemically administered interventions or allowed combination therapies, making it difficult to isolate the independent clinical contribution of the herbal intervention itself.

In contrast, the present study specifically examined herbal retention enema as a standalone intervention, thereby minimizing the confounding effects of concomitant Western medications. This approach enables a clearer assessment of the intrinsic therapeutic effects of TCM when administered via the rectal route. In addition, whereas previous reviews of oral TCM frequently noted gastrointestinal adverse effects as a limitation, the findings of this review suggest that retention enema may be associated with fewer gastrointestinal complaints, potentially offering a better-tolerated option for certain patients.

A further distinguishing aspect of this study is its focus on localized drug delivery. Given the anatomical proximity of the rectum to the recto-uterine pouch (Douglas pouch), a common site of endometriotic lesions, rectal administration may facilitate relatively higher local exposure of active compounds within the pelvic cavity. This localized delivery concept is consistent with the larger effect size observed for retention enema compared with oral administration (OR 7.13), although the underlying pharmacokinetic mechanisms remain to be elucidated.

### 4.3. Limitation of the Study

Several important limitations should be considered when interpreting the findings of this study.

First, concerns related to the methodological quality and risk of bias of the included primary studies should be acknowledged. In most studies, reporting of random sequence generation and allocation concealment was insufficient, resulting in a judgment of some concerns regarding bias arising from the randomization process. Moreover, due to the nature of herbal retention enema, blinding of participants and practitioners was not feasible, which may have introduced bias in outcome measurement, particularly for subjective outcomes such as pain scores and total effective rate. In addition, the absence of prospectively registered study protocols in many trials raises concerns about potential selective reporting bias.

Second, there was substantial structural heterogeneity in both interventions and outcome measures. Across the included studies, the herbal formulations used for retention enema, as well as treatment temperature, retention time, frequency, and duration, varied considerably, limiting the standardization of the intervention.

Importantly, the pooled estimates relied heavily on the total effective rate, which is a non-validated composite endpoint frequently reported in Chinese trials. Because definitions and assessment criteria varied across studies, and this measure is not routinely used in international endometriosis research, the clinical validity and generalizability of these pooled findings are limited and require cautious interpretation. Variations in pain assessment tools and in the timing and reporting of biomarker measurements also constrained quantitative synthesis for certain outcomes.

Third, the generalizability of the findings and the availability of long-term evidence remain limited. All included studies were conducted in China, making it difficult to fully exclude the possibility of regional bias and publication bias. Therefore, the current evidence should be regarded as preliminary, and confirmation in independent trials conducted in other geographical and healthcare settings is required before broader clinical conclusions can be drawn. In addition, the relatively small sample sizes of the included trials may have resulted in insufficient statistical power for some outcomes, particularly pregnancy rate and biomarker analyses. Finally, as most studies were limited to short-term follow-up, evidence regarding the long-term sustainability of treatment effects and the potential role of herbal retention enema in preventing disease recurrence remains insufficient. The implication of this data gap is critical, as endometriosis is a chronic, relapsing condition that often necessitates lifelong management. Without longitudinal data, it remains unclear whether the symptomatic relief is maintained after treatment cessation or if this localized therapy effectively addresses the high recurrence rates that characterize the disease—a primary clinical challenge in endometriosis care.

### 4.4. Implications for Future Research

To strengthen the evidence base for herbal retention enema in endometriosis management, future research should address several key methodological and clinical considerations.

First, enhanced methodological rigor is essential. Future clinical trials should adhere to established reporting standards, such as the CONSORT statement, and clearly document procedures for random sequence generation, allocation concealment, and blinding where feasible. Prospective registration in international clinical trial registries is also strongly recommended to reduce the risk of publication and selective reporting biases.

Second, there is a critical need for the standardization of outcome measures. Future studies should consider moving beyond the traditional total effective rate and prioritize internationally validated, patient-centered outcome tools. Future trials should prioritize internationally accepted endpoints such as the Visual Analog Scale (VAS) for pain, the Endometriosis Health Profile-30(EHP-30) and disease-specific quality-of-life instruments. Standardizing the timing of assessments and the selection of biological markers, including CA-125 and inflammatory cytokines, would further enhance comparability across studies and facilitate more robust quantitative synthesis.

Third, adequately powered, multi-center randomized controlled trials with longer follow-up periods are warranted. Given the chronic and recurrent nature of endometriosis, studies extending beyond 6–12 months are needed to evaluate the sustained effects of herbal retention enema on clinically meaningful outcomes, including pregnancy rates and recurrence prevention. Moreover, independent international trials conducted outside China are essential to confirm these findings and improve their generalizability across diverse populations.

Finally, complementary mechanistic and pharmacokinetic studies are needed to better understand the biological basis of herbal retention enema. Future research may explore how active compounds administered rectally are distributed within the pelvic region and how such localized exposure influences the pelvic inflammatory microenvironment. Elucidating these mechanisms would provide a stronger scientific rationale for the integration of this traditional intervention into contemporary gynecological practice.

## 5. Conclusions

This systematic review and meta-analysis provide the first comprehensive synthesis of the clinical efficacy and safety of Traditional Chinese Medicine herbal retention enema for endometriosis. Based on evidence from nine randomized controlled trials involving 614 participants, herbal retention enema was associated with greater overall clinical effectiveness compared with Western medicine (OR = 2.87) and oral herbal medicine (OR = 7.13), as well as a significant reduction in dysmenorrhea severity. A trend toward improved pregnancy outcomes was also observed (*p* = 0.05). However, the available evidence remains preliminary.

In terms of safety, herbal retention enema demonstrated a relatively favorable tolerability profile, with no serious adverse events reported and fewer systemic adverse effects compared with hormonal therapies. Moreover, the consistency of procedural characteristics, such as enema volume, and retention time across studies suggests that this localized delivery approach may partly contribute to the observed clinical benefits by enhancing pelvic exposure while limiting systemic effects.

Despite these promising findings, the formulation of our clinical recommendations was heavily weighted against the identified methodological limitations. Specifically, concerns regarding randomization procedures and the lack of blinding may have inflated subjective outcomes such as pain relief. Furthermore, the lack of long-term follow-up has significant clinical implications; as endometriosis is a chronic and relapsing condition, it remains unclear whether the symptomatic improvements are sustained after treatment cessation or if this therapy effectively reduces recurrence rates—a primary challenge in endometriosis management.

In addition, because all included trials were conducted exclusively in China, the generalizability of these findings to other populations remains uncertain. While herbal retention enema may have potential as a complementary approach, the current evidence is preliminary and of low certainty. Well-designed randomized controlled trials with rigorous methodology and independent international confirmation are required before firm clinical recommendations can be made.

## Figures and Tables

**Table 1:** Characteristics and clinical outcomes of the included studies.

First Author (Year)	Diagnosis Criteria	Sample Size (Exp/Con)	Intervention	Comparator	Outcome	Result (Exp vs. Con)	F/U	Adverse Event (Exp vs. Con)
Gao (2013) [17]	Laparoscopic diagnosis	42:40	Retention enema therapy	Gestrinone 2.5 mg, twice/week	① Total effective rate ② Recurrence rate ③ Pregnancy rate	① 78.6% vs. 55.0% ^a^ ② 4.8% vs. 20.0% ^a^ ③ 58.3% vs. 27.2% ^a^	12 months	0 vs. 23
Qu (2013) [18]	Integrated Traditional Chinese and Western Medicine diagnostic criteria	24:24	Retention enema therapy	Danazol 200 mg, twice/day	① Total effective rate ② Pregnancy rate ③ Dysmenorrhea score ④ Pelvic mass size ⑤ Serum markers	① 87.5% vs. 91.6% ^d^ ② 33.3% vs. 18.2% ^d^ ③ 9.24 ± 2.18 → 6.31 ± 4.19 vs. 8.19 ± 2.64 → 2.86 ± 3.99 ^a^ ④ 40.83 ± 30.22 → 14.99 ± 21.70 vs. 45.52 ± 31.40 → 18.63 ± 24.87 ^b^ ⑤-1. TXB_2_ (pg/mL) 154.63 ± 149.2 → 91.30 ± 84.09 vs. 169.78 ± 153.3 → 96.59 ± 87.14 ^a^ ⑤-2. 6-keto PGF1α (pg/mL) 119.17 ± 117.23 → 75.23 ± 71.95 vs. 134.89 ± 126.18 → 81.03 ± 74.83 ^a^ ⑤-3. CA-125 (U/mL) 56.72 ± 30.11 → 49.91 ± 19.39 vs. 54.17 ± 31.85 → 47.17 ± 19.47 ^a^	3 months	3 vs. 7
Dang (2014) [19]	r-AFS stage II or higher	35:35	Retention enema therapy	Gestrionone 2.5 mg, twice/week	① Total effective rate ② Symptom prevalence ③ Serum markers	① 82.4% vs. 83.9% ^d^ ②-1. Dysmenorrhea 18 → 5 vs. 20 → 7 ^d^ ②-2. Menorrhagia 12 → 4 vs. 13 → 3 ^d^ ②-3. Infertility 14→5 vs. 12→6 ^d^ ③-1. IL-1β (pg/mL) 33.16 ± 3.25 → 10.48 ± 2.34 vs. 34.15 ± 3.48 → 12.76 ± 3.17 ^a^ ③-2. TNF-α (pg/mL) 83.22 ± 14.26 → 34.78 ± 11.47 vs. 82.95 ± 13.23 → 35.49 ± 12.15 ^a^ ③ NF-κB p56 (ng/L) 0.614 ± 0.042 → 0.273 ± 0.037 vs 0.667 ± 0.056 → 0.280 ± 0.044 ^a^	N.R.	3 vs. 21
Li (2018) [20]	Integrated Traditional Chinese and Western Medicine diagnostic criteria	40:40	Retention enema therapy	Herbal medicine (oral) 3 capsules, three times/day	Total effective rate	95.0% vs. 77.5% ^a^	N.R.	N.R.
Chang (2022) [21]	Clinical Practice Guidelines for diagnosis	38:38	Retention enema therapy	Gestrinone 2.5 mg, twice/week	① Total effective rate ② Symptom prevalence ③ Serum hormone level	① 73.7% vs. 92.1% ^a^ ②-1. Dysmenorrhea 3.26 ± 1.34 → 0.72 ± 0.12 vs. 3.32 ± 1.11 → 1.82 ± 0.43 ^c^ ②-2. Breast pain 3.57 ± 0.88 → 0.62 ± 0.22 vs. 3.42 ± 0.91 → 1.71 ± 0.53 ^c^ ②-3. Anal swelling 3.45 ± 0.74 → 0.62 ± 0.28 vs. 3.32 ± 0.61 → 1.33 ± 0.31 ^c^ ③-1. FSH (IU/L) 9.77 ± 1.74 → 5.11 ± 1.66 vs. 9.85 ± 1.92 → 7.33 ± 2.41 ^c^ ③-2. E2 (pg/mL) 42.15 ± 3.89 → 12.31 ± 0.62 vs. 43.31 ± 3.62 → 17.43 ± 2.81 ^c^	N.R.	N.R.
Zhou (2012) [22]	N.R.	46:34	Retention enema therapy	Gestrinone 25 mg, twice/week	① Total effective rate ② Dysmenorrhea score	① 93.4% vs. 76.5% ^a^ ② 11.28 ± 3.24 → 4.14 ± 1.12 vs. 11.15 ± 3.18 → 7.12 ± 3.21 ^a^	N.R.	0 vs. 11
Zeng (2018) [23]	N.R.	30:30	Retention enema therapy	Herbal medicine PO, 3 times/day	① Total effective rate ② Symptom score ③ Cyst diameter	① 96.67% vs. 73.33% ^a^ ② 8.20 ± 3.17 → 0.87 ± 1.07 vs. 7.97 ± 2.77 → 2.00 ± 1.93 ^b^ ③ 30.18 ± 5.43 → 29.88 ± 5.01 (mm) vs. 27.73 ± 4.46 → 27.47 ± 4.22 (mm) ^a^	N.R.	N.R.
Zhang (2007) [24]	Ultrasonography	40:36	Retention enema therapy	Danazol 200 mg, twice/day	Total effective rate	85.0% vs. 86.7% ^d^	N.R.	0 vs. 10
Zou (2017) [25]	N.R.	21:21	Retention enema therapy	Danazol 200 mg, twice/day	Total effective rate	95.24% vs. 71.43% ^a^	N.R.	N.R.

Abbreviation: Exp, Experiment; Con, Control; F/U, follow up; N.R.; Not reported; PO, per os; r-AFS, revised American Fertility Society classification; TXB_2_, thromboxane B_2_; 6-keto PGF_1_α, 6-keto-prostaglandin F_1_α; CA-125, cancer antigen 125; IL-1β, interleukin-1 beta; TNF-α, tumor necrosis factor alpha; NF-κB, nuclear factor kappa B; FSH, follicle-stimulating hormone; E2, estradiol, a, *p* < 0.05; b, *p* < 0.01; c, *p* < 0.001; d, *p* > 0.05.

**Table 2:** Characteristics of herbal retention enema interventions.

First Author (Year)	Name of Prescription	Herbal Composition	Dosage (mL)	Retention Time	Frequency	Total Duration	Additional Details
Gao (2013) [17]	Quyusanjie Fang	*Angelica sinensis* (*Angelica sinensis* (Oliv.) Diels) (15 g), *Leonuri Herba* (*Leonurus japonicus* Houtt.) (15 g), *Achyranthis Bidentatae Radix* (*Achyranthes bidentata Blume*) (12 g), *Curculiginis Rhizoma* (*Curculigo orchioides* Gaertn.) (12 g), *Eucommiae Cortex* (*Eucommia ulmoides* Oliv.) (12 g), *Curcumae Rhizoma* (*Curcuma phaeocaulis* Valeton) (10 g), *Persicae Semen* (*Prunus persica* (L.) Batsch) (10 g), *Carthami Flos* (*Carthamus tinctorius* L.) (10 g), *Salviae Miltiorrhizae Radix* (*Salvia miltiorrhiza* Bunge) (10 g), *Paeoniae Radix Rubra* (*Paeonia lactiflora* Pall.) (10 g), *Curcumae Radix* (*Curcuma wenyujin* Y.H.Chen & C.Ling) (9 g), *Artemisiae Argyi Folium (Artemisia argyi* H.Lév. & Vaniot) (9 g), *Notoginseng Radix (Panax notoginseng* (Burkill) F.H.Chen) (6 g)	100	More than 30 min	Once a day	6 months	Treatment was initiated 7 days after surgery. Discontinued during menstruation.
Qu (2013) [18]	Not reported	*Epimedii Herba* (*Epimedium brevicornum* Maxim.) (15 g), *Cuscutae Semen* (*Cuscuta chinensis* Lam.) (15 g), *Eupolyphaga seu Steleophaga* (*Eupolyphaga sinensis* Walker) (15 g), *Paeoniae Radix Alba* (*Paeonia lactiflora* PalL.) (15 g), *Fritillariae Thunbergii Bulbus* (*Fritillaria thunbergii* Miq.) (15 g), *Angelica sinensis* (*Angelica sinensis* (Oliv.) Diels) (12 g), *Curcumae Rhizoma* (*Curcuma phaeocaulis* Valeton) (10 g), *Curcumae Radix* (*Curcuma wenyujin* Y.H.Chen & C.Ling) (10 g), *Persicae Semen* (*Prunus persica* (L.) Batsch) (10 g), *Carthami Flos* (*Carthamus tinctorius* L.) (10 g), *Trogopterorum Faeces* (feces of *Trogopterus xanthipes* Milne-Edwards) (10 g), *Typhae Pollen* (*Typha angustifolia* L.) (10 g), *Cyperi Rhizoma* (*Cyperus rotundus* L.) (10 g), *Manis Squama* (scales of *Manis pentadactyla* Linnaeus) (4 g)	180	Not reported	Once a day	3 months	Discontinued during menstruation.
Dang (2014) [19]	Not reported	*Epimedii Herba* (*Epimedium brevicornum* Maxim.) (15 g), *Cuscutae Semen* (*Cuscuta chinensis* Lam.) (15 g), *Eupolyphaga seu Steleophaga* (*Eupolyphaga sinensis* Walker) (15 g), *Paeoniae Radix Alba* (*Paeonia lactiflora* PalL.) (15 g), *Fritillariae Thunbergii Bulbus* (*Fritillaria thunbergii* Miq.) (15 g), *Angelica sinensis* (*Angelica sinensis* (Oliv.) Diels) (12 g), *Curcumae Rhizoma* (*Curcuma phaeocaulis* Valeton) (10 g), *Curcumae Radix* (*Curcuma wenyujin* Y.H.Chen & C.Ling) (10 g), *Persicae Semen* (*Prunus persica* (L.) Batsch) (10 g), *Carthami Flos* (*Carthamus tinctorius* L.) (10 g), *Trogopterorum Faeces* (feces of *Trogopterus xanthipes* Milne-Edwards) (10 g), *Typhae Pollen* (*Typha angustifolia* L.) (10 g), *Cyperi Rhizoma* (*Cyperus rotundus* L.) (10 g), *Manis Squama* (scales of *Manis pentadactyla* Linnaeus) (5 g)	180	Not reported	Once a day	6 months	Discontinued during menstruation.
Li (2018) [20]	Funing Tang	*Paeoniae Radix Rubra* (*Paeonia lactiflora* PalL.) (15 g), *Curcumae Rhizoma* (*Curcuma phaeocaulis* Valeton) (10 g), *Persicae Semen* (*Prunus persica* (L.) Batsch) (10 g), *Cinnamomi Ramulus* (*Cinnamomum cassia* (L.) J.Presl) (10 g), *Moutan Cortex* (*Paeonia suffruticosa* Andrews) (10 g), *Achyranthis Bidentatae Radix* (*Achyranthes bidentata* Blume) (10 g), *Curcumae Radix* (*Curcuma wenyujin* Y.H.Chen & C.Ling) (9 g), *Gleditsiae Spina* (*Gleditsia sinensis* Lam.) (9 g)	150	More than 30 min	Once a day	3 menstrual cycles	Initiated after the end of menstruation Discontinued during menstruation
Chang (2022) [21]	Huayu Xiaozheng Decoction	*Astragali Radix* (*Astragalus membranaceus* (Fisch.) Bunge) (25 g), *Impatientis Semen* (*Impatiens balsamina* L.) (10 g), *Typhae Pollen* (*Typha angustifolia* L.) (10 g), *Corydalis Rhizoma* (*Corydalis yanhusuo* W.T.Wang) (10 g), *Achyranthis Bidentatae Radix* (*Achyranthes bidentata* Blume) (10 g), *Cinnamomi Ramulus* (*Cinnamomum cassia* (L.) J.Presl) (10 g)*, Eupolyphaga seu Steleophaga* (*Eupolyphaga sinensis* Walker) (6 g), *Hirudo* (*Hirudo nipponia* Whitman) (5 g)	100	Not reported	Once a day	6 months	Treatment was initiated 7 days after surgery. Administered from 10 days before menstruation to menstruation onset.
Zhou (2012) [22]	Yiwei Tongjingling	*Salviae Miltiorrhizae Radix* (*Salvia miltiorrhiza* Bunge) (30 g), *Curcumae Rhizoma* (*Curcuma phaeocaulis* Valeton) (15 g), *Curcumae Radix* (*Curcuma wenyujin* Y.H.Chen & C.Ling) (15 g), *Astragali Radix* (*Astragalus membranaceus* (Fisch.) Bunge) (15 g), *Spatholobi Caulis* (*Spatholobus suberectus* Dunn) (15 g), *Psoraleae Fructus* (*Psoralea corylifolia* L.) (12 g), *Cinnamomi Ramulus* (*Cinnamomum cassia* (L.) J.Presl) (10 g), *Myrrha* (*Commiphora myrrha* (Nees) EngL.) (10 g), *Rhei Radix et Rhizoma* (*Rheum palmatum* L.) (10 g), *Vespae Nidus* (*nest of Polistes* spp.) (10 g)	100	More than 2 h	Once a day	3 months	Initiated 3–5 days after menstruation onset. Discontinued during menstruation.
Zeng (2018) [23]	Not reported	*Cinnamomi Ramulus* (*Cinnamomum cassia* (L.) J.Presl) (30 g), *Curcumae Rhizoma* (*Curcuma phaeocaulis* Valeton) (30 g), *Corydalis Rhizoma* (*Corydalis yanhusuo* W.T.Wang) (30 g), *Artemisiae Anomalae Herba* (*Artemisia anomala* S.Moore) (30 g), *Curcumae Radix* (*Curcuma wenyujin* Y.H.Chen & C.Ling) (30 g), *Paeoniae Radix Rubra* (*Paeonia lactiflora* PalL.) (30 g), *Cnidii Fructus* (*Cnidium monnieri* (L.) Cusson) (30 g), *Cyperi Rhizoma* (*Cyperus rotundus* L.) (30 g), *Linderae Radix* (*Lindera aggregata* (Sims) Kosterm.) (30 g), *Lycopi Herba* (*Lycopus lucidus* Turcz. ex Benth.) (30 g), *Sargentodoxae Caulis* (*Sargentodoxa cuneata* (Oliv.) Rehder & E.H.Wilson) (30 g), *Gleditsiae Spina* (*Gleditsia sinensis* Lam.) (30 g), *Lonicerae Japonicae Caulis* (*Lonicera japonica* Thunb.) (30 g), *Spatholobi Caulis* (*Spatholobus suberectus* Dunn) (30 g), *Speranskiae Tuberculatae Herba* (*Speranskia tuberculata* (Bunge) BailL.) (30 g), *Ephedrae Herba* (*Ephedra sinica* Stapf) (20 g), *Morindae Officinalis Radix* (*Morinda officinalis* F.C.How) (20 g), *Poria* (*Wolfiporia cocos* (Schwein.) Ryvarden & Gilb.) (20 g), *Asari Radix et Rhizoma* (*Asarum heterotropoides* F.Schmidt) (15 g), *Olibanum* (*Boswellia carterii* Birdw.) (15 g), *Hirudo* (*Hirudo nipponia* Whitman) (15 g), *Myrrha* (*Commiphora myrrha* (Nees) EngL.) (15 g), *Gardeniae Fructus* (*Gardenia jasminoides* J.Ellis) (15 g)	100	Not reported	Once a day	3 months	Initiated after the end of menstruation. Discontinued during menstruation
Zhang (2007) [24]	Qingyi Tang	*Spatholobi Caulis* (*Spatholobus suberectus* Dunn) (30 g), *Salviae Miltiorrhizae Radix* (*Salvia miltiorrhiza* Bunge) (20 g), *Patriniae Herba* (*Patrinia scabiosaefolia* Fisch. ex Trevir.) (15 g), *Corydalis Rhizoma* (*Corydalis yanhusuo* W.T.Wang) (12 g), *Paeoniae Radix Rubra* (*Paeonia lactiflora* PalL.) (12 g), *Angelica sinensis* (*Angelica sinensis* (Oliv.) Diels) (12 g), *Fritillariae Thunbergii Bulbus* (*Fritillaria thunbergii* Miq.) (10 g), *Persicae Semen* (*Prunus persica* (L.) Batsch) (10 g), *Olibanum* (*Boswellia carterii* Birdw.) (6 g), *Myrrha* (*Commiphora myrrha* (Nees) EngL.) (6 g)	100	More than 4 h	Once a day	3 months	Discontinued during menstruation.
Zou (2017) [25]	Neiyixiao	*Epimedii Herba* (*Epimedium brevicornum* Maxim.) (12 g), *Cuscutae Semen* (*Cuscuta chinensis* Lam.) (12 g), *Eupolyphaga seu Steleophaga* (*Eupolyphaga sinensis* Walker) (12 g), *Paeoniae Radix Alba* (*Paeonia lactiflora* PalL.) (12 g), *Fritillariae Thunbergii Bulbus* (*Fritillaria thunbergii* Miq.) (12 g), *Angelica sinensis* (*Angelica sinensis* (Oliv.) Diels) (10 g), *Curcumae Rhizoma* (*Curcuma phaeocaulis* Valeton) (8 g), *Carthami Flos* (*Carthamus tinctorius* L.) (8 g), *Persicae Semen* (*Prunus persica* (L.) Batsch) (8 g), *Curcumae Radix* (*Curcuma wenyujin* Y.H.Chen & C.Ling) (8 g), *Typhae Pollen* (*Typha angustifolia* L.) (8 g), *Trogopterorum Faeces* (feces of *Trogopterus xanthipes* Milne-Edwards) (8 g), *Cyperi Rhizoma* (*Cyperus rotundus* L.) (8 g)	180	Not reported	Once a day	3 months	Discontinued during menstruation.

## Data Availability

No new data were created or analyzed in this study.

## Supplementary Materials

The following supporting information can be downloaded at: https://www.mdpi.com/article/10.3390/ph19020279/s1, Additional File S1. Search terms used in each database and results, Additional File S2. PRISMA checklist [31].

## Abbreviations

The following abbreviations are used in this manuscript:

6-keto PGF_1_α	6-keto-prostaglandin F_1_α
CA-125	Cancer antigen 125
CAM	Complementary and Alternative Medicine
Con	Control
E2	Estradiol
Exp	Experiment
F/U	Follow up
FSH	Follicle-stimulating hormone
IL-1β	Interleukin-1 beta
NF-κB	Nuclear factor kappa B
N.R.	Not reported
OR	Odds ratio
PO	Per os
RCT	Randomized Controlled Trial
RoB	Risk of Bias
RR	Risk ratio
r-AFS	revised American Fertility Society classification
TNF-α	Tumor necrosis factor alpha
TXB_2_	Thromboxane B_2_
