# Supplementary material for: Efficacy and Safety of Traditional Chinese Medicine Retention Enema for Endometriosis: A Systematic Review and Meta-Analysis
Source: Pharmaceuticals (Basel). 2026 Feb 6;19(2):279. doi: 10.3390/ph19020279 (PMC12944565; doi:10.3390/ph19020279)
Supplement: Supplementary file 1 [file pharmaceuticals-19-00279-s001.zip › Supplementary File S2. Search terms used in each database and results.pdf]

Supplementary File S2. Search terms used in each database and

results Medline via PubMed

	Searches	Results
#1	Endometriosis[Mesh] OR Endometrioma[Mesh] OR Endometriosis[TIAB] OR Endometrioma[TIAB]	27,463
#2	Enema[Mesh] OR Enema[TIAB]	7384
#3	#1 AND #2	42

Embase via Elsevier

	Searches	Results
#1	Endometriosis/exp OR Endometrioma/exp OR Endometriosis:ab,ti OR Endometrioma:ab,ti	54,647
#2	Enema/exp OR Enoma:ab,ti	12,579
#3	#1 AND #2	312

CINAHL via EBSCO

	Searches	Results
#1	Endometriosis	10,375
#2	Endometrioma	1,044
#3	Enema	2,272
#4	Retention enema	89
#5	Herbal enema	59
#6	#1 AND (#3 OR #4 OR #5)	27
#7	#2 AND (#3 OR #4 OR #5)	0

Web of Science

	Searches	Results
#1	Endometriosis	34,786
#2	Endometrioma	2,731
#3	Enema	9,185
#4	#1 AND #2	107
#5	#1 AND #3	4

Chinese medical databases – China National Knowledge Infrastructure (CNKI), Wanfang Data

	Searches	Results
#1	子宫内膜异位症/SU OR Endometriosis/SU	52,372
#2	子宫内膜瘤/SU OR Endometrioma/SU	20,234
#3	保留灌肠/SU OR Enema/SU	49,259
#4	#1 AND #3	177
#5	#2 AND #3	0

Korean medical databases – Korean Studies Information Service System (KISS), Research Information Sharing Service (RISS), Oriental Medicine Advanced Searching Integrated System (OASIS), Korea Citation Index (KCI), and the Korean Medical Database (KMbase)

	Searches	Results
#1	자궁내막증 OR 자궁내막종	349
#2	관장 OR 보류관장	1,637
#3	#1 AND #2	0
